# Comparative analysis of newly identified rodent arteriviruses and porcine reproductive and respiratory syndrome virus to characterize their evolutionary relationships

**DOI:** 10.3389/fvets.2023.1174031

**Published:** 2023-04-03

**Authors:** Zhuang-Yan Zhao, De Yu, Chun-Miao Ji, Qiankun Zheng, Yao-Wei Huang, Bin Wang

**Affiliations:** ^1^Department of Veterinary Medicine, Zhejiang University, Hangzhou, Zhejiang, China; ^2^Guangdong Laboratory for Lingnan Modern Agriculture, College of Veterinary Medicine, South China Agricultural University, Guangzhou, China; ^3^DELISI GROUP Co., LTD., Delisi Industrial Park, Weifang, China

**Keywords:** rodent arterivirus, porcine reproductive and respiratory syndrome virus (PRRSV), genome, origin, evolution

## Abstract

Porcine reproductive and respiratory syndrome virus (PRRSV) has caused huge economic losses for the global pig industry, but its origins and evolution remain a mystery. In 2018, the genome sequences of seven arteriviruses isolated from rodents were determined, and here we publish new analysis showing that they may be ancestors of PRRSV. The sequence similarity of these viruses to PRRSV was ~60%, with shared genome organization and other characteristics, such as slippery sequences and C-rich motifs in nsp2, and a transactivated protein sequence in nsp1β. Codon usage basis analysis showed that PRRSV was closer to these rodent arteriviruses than lactate dehydrogenase-elevating virus (LDV) and they were both under pressure of natural selection. Evolutionary analysis revealed that four of the rodent arteriviruses shared the same genus with PRRSV, and were more closely related to PRRSV-2 than PRRSV-1. In addition to this, they all appeared earlier than PRRSV according to evolutionary modeling, and we speculate that they represent an intermediate step in the origin of PRRSV by arterivirus transmission from rodents to swine. Our in-depth analysis furthers our understanding of arteriviruses, and will serve as the basis for subsequent exploration of the evolution of PRRSV and other arteriviruses.

## Introduction

Arteriviruses can infect domestic and wild animals, causing a variety of diseases (1). Arteriviruses (order *Nidovirales*; family *Arteriviridae*) have a positive-sense, single-stranded RNA genome that ranges from 12 to 16 kb (2). Among them, equine arteritis virus (EAV), lactate dehydrogenase-elevating virus (LDV) and simian hemorrhagic fever virus (SHFV) were first isolated separately in 1953, 1960, and 1964, respectively, while porcine reproductive and respiratory syndrome virus (PRRSV) was first recognized in the late 1980s (3–6). Emerging arteriviruses have been discovered in recent years, such as the highly divergent wobbly possum disease virus (WPDV) in New Zealand and some rodent arteriviruses in China (7, 8).

PRRSV has had the greatest economic impact of all arteriviruses, causing reproductive problems in pregnant sows including abortion, stillbirth and mummified fetuses as well as respiratory disease such as pneumonia and dyspnea in piglets (9, 10). PRRSV is divided into two genotypes that share only 60% sequence similarity: PRRSV-1 is mainly distributed in European countries; PRRSV-2 is mainly distributed in North America and Asia (11, 12). Their prototypes, Lelystad virus and VR-2332, respectively, were isolated separately in the Netherlands and the United States almost simultaneously (13, 14). Subsequently, outbreaks of severe disease occurred in the United States in 1996 and 2001, and in China in 2006 (6, 15–17).

Though the exact ancestor of PRRSV is still unknown, some have speculated it to be LDV; however, several strains of arterivirus have recently been isolated from rodents in China, with a much closer evolutionary relationship to PRRSV than LDV, leading to speculation that they are likely the original ancestor of PRRSV (8). Therefore, the aim of this study was to investigate this newly discovered evolutionary relationship by comparing the genome organization and codon usage bias in details, providing further insight into the origin of PRRSV.

## Materials and methods

### Genetic material

The complete sequences of 43 arteriviruses were downloaded from GenBank (http://www.ncbi.nlm.nih.gov); detailed information about the viruses is listed in Supplementary Table S1.

### Codon usage analysis

Different aspects of codon usage in the arterivirus coding sequences (CDS) were analyzed: the frequency of nucleotides (A%, C%, U%, and G%); G+C content (GC); G+C content at the first, second or third position, (GC1, GC2, GC3, respectively); the frequency of nucleotides G+C at the third synonymous codon positions (GC3s); codon adaptation index (CAI), representing the fitness coefficient of all codons encoding the protein in the case of using optimal codons; effective number of codons (ENC), reflecting the degree to which codons deviate from random selection (usually low-expressed genes have a weaker codon usage bias and a larger ENC value).

The nucleotide content of each arterivirus coding sequence was calculated using the CAI calculator website (http://genomes.urv.es/CAIcal/), and CAI and ENC values were calculated using the program CodonW 1.4.2.

### Neutrality plot analysis

Neutrality plot analysis is an analytical method for evaluating the use of codons, and it reflects the factors that affect codon usage bias. A scatter plot was drawn with GC12 as the vertical coordinate and GC3 as the abscissa coordinate of each gene, and the correlation between the two was analyzed. If the regression coefficient was >0 and the correlation coefficient >0.75, it meant that GC3 was significantly related to GC12, further illustrating that the codons in three positions have the same variation pattern and mutation is the main factor of codon usage bias. Conversely, if the correlation between GC3 and GC12 was not significant, it indicated that the variation patterns of the codons in three positions are quite different, i.e., the codon usage bias is mainly affected by natural selection. The scatter plot was drawn by Graphpad Prism 8.0.2.

### ENC-GC3s plot analysis

The ENC-GC3s plot was drawn with the ENC values plotted against the GC3s values, and the theoretical ENC values of each gene were calculated according to the formula for drawing the standard curves, with GC3s as the abscissa and the theoretical ENC values as the ordinate. A gene lying on or near the standard curve indicates that the codon usage bias is only affected by mutation and has no selection pressure, whereas distribution far away from the standard curve means the codon usage bias is mainly affected by natural selection factors. The standard ENC values were calculated using the formula:


$$\begin{array}{l} {\text{ENC}}_{\text{expected}}=2+\text{S}+\frac{29}{\left({\text{S}}^{2}+{\left(1-\text{S}\right)}^{2}\right)} \end{array}$$

where “s” represents the given GC3s value.

### Parity rule 2 plot (PR2-plot) analysis

In this plot, G3/(G3 + C3) was compared to A3/(A3 + U3) to analyze the base composition on the nucleotide of the third codon, so as to explore the influence of mutation and natural selection on the codon usage bias. The midpoint 0.5 in Figure 3C represents A = U and G = C, indicating no bias toward mutation or selection effect between the two complementary strands of a gene; the vector from the center point to other site shows the degree and direction of bias in the gene. The neutral theory proves that if codon usage bias is only affected by genetic mutations, then the usage frequency of the four bases will be equal, and a relatively equal distribution is expected to be shown in the plot. The scatter plot was drawn by Graphpad Prism 8.0.2.

### Relative synonymous codon usage (RSCU) analysis

RSCU represents the ratio of the actual usage value of the codon to the theoretical use value. When RSCU is <1 or >1, it means the usage frequency of the codon is lower or higher, respectively, than that of other synonymous codons; RSCU = 1 considers that the codon has no preference. The codon usage pattern heatmap was drawn by TBtools (18), and RSCU values were calculated as follows:


$$\text{RSCU}=\frac{{\text{x}}_{\text{ij}}}{\sum_{\text{j}}^{\text{ni}}{\text{x}}_{\text{ij}}}{\text{n}}_{\text{i}}$$

### Phylogenetic analysis

Sequence alignment of the complete genomes and selected genes including RNA-dependent RNA polymerase (RdRp), helicase (Hel), 3C-like protease (3CLpro) and nucleocapsid (N) was performed using MAFFT v 7.475 (19). Maximum-likelihood (ML) phylogenetic tree construction was performed with 1,000 bootstraps using IQ-tree v 1.6.12 (20), and the best-fitting nucleotide substitution model was calculated automatically by the program. Finally, the results were visualized using iTOL (http://itol.embl.de/).

### Origin and evolutionary analysis

In order to investigate the relationship between the rodent arteriviruses and PRRSV, a time-scaled phylogenetic tree of the Hel gene was constructed. The evolutionary rate was estimated using BEAST v 1.10.4 with a separate GTR+F+G4 nucleotide substitution model analyzed by PhyloSuite v 1.2.2, and the uncorrelated lognormal relaxed constant clock model selected by both path sampling and stepping-stone sampling procedures (Table 1) (21–23). We operated MCMC duplicate runs of 1 billion states each with sampling every 100,000 steps, and then examined the result using Tracer v1.6 to ensure the effective sample size (ESS) values of all estimated parameters was >200. The final maximum clade credibility (MCC) tree was generated by TreeAnnotator v 1.10.4 and visualized in Figtree v1.4.4 after discarding the first 10% of the samples.

**Table 1:** The marginal likelihoods estimated of molecular clock models and coalescent models.

**Model of rate variation**	**Coalescent tree prior**	**Log marginal likelihood**	**Rank**
Strict clock	Constant size	−21643.0146	4
Strict clock	Bayesian skyline	−21641.4570	3
**Uncorrelated lognormal relaxed clock**	**Constant size**	**−21485.3313**	**1**
Uncorrelated lognormal relaxed clock	Bayesian skyline	−21488.8779	2

The best-fitting combination of molecular clock models and coalescent tree prior are indicated in bold font.

## Results

### Genome organization and characteristics

**Figure 1:**
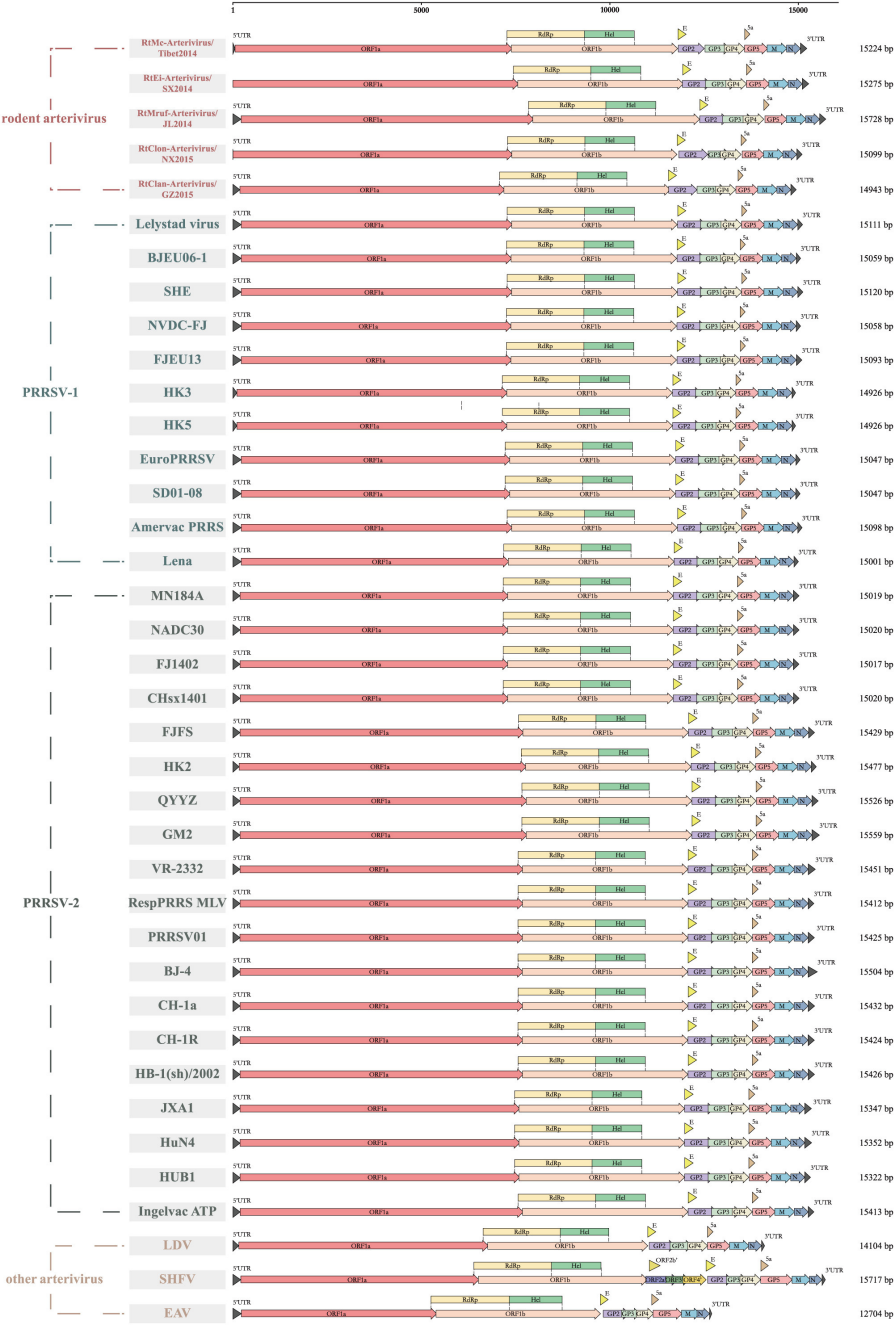
Genome organization of different arteriviruses. Every ORF is marked to show the genomic differences among them. ORF1a, ORF1b, GP2, envelope (E), GP3, GP4, 5a, GP5, membrane (M), nucleocapsid (N) are represented by boxes of different colors.

Rodent arteriviruses have single-stranded, positive-sense polycistronic RNA genomes with similar organization to the other arteriviruses (Figure 1). Their genomes encode at least 11 known open reading frames (ORF) with a 5′ cap and a 3′ poly-A tail, in the order of 5′-polyprotein (pp) 1a, pp1b, GP2, envelope (E), GP3, GP4, 5a, GP5, membrane (M) and N-3′. The first two ORFs occupy two-thirds of the genomes and use a −1 programmed ribosomal frameshifting (-1PRF) strategy to encode at least 13 non-structural proteins (nsps). The size and the GC content of the genomes of seven rodent arterivirus are shown in Supplementary Table S1, where two of them, RtDs-Arterivirus-4/IM2014 and RtDs-Arterivirus-1/IM2014, did not detect the full length.

**Figure 2:**
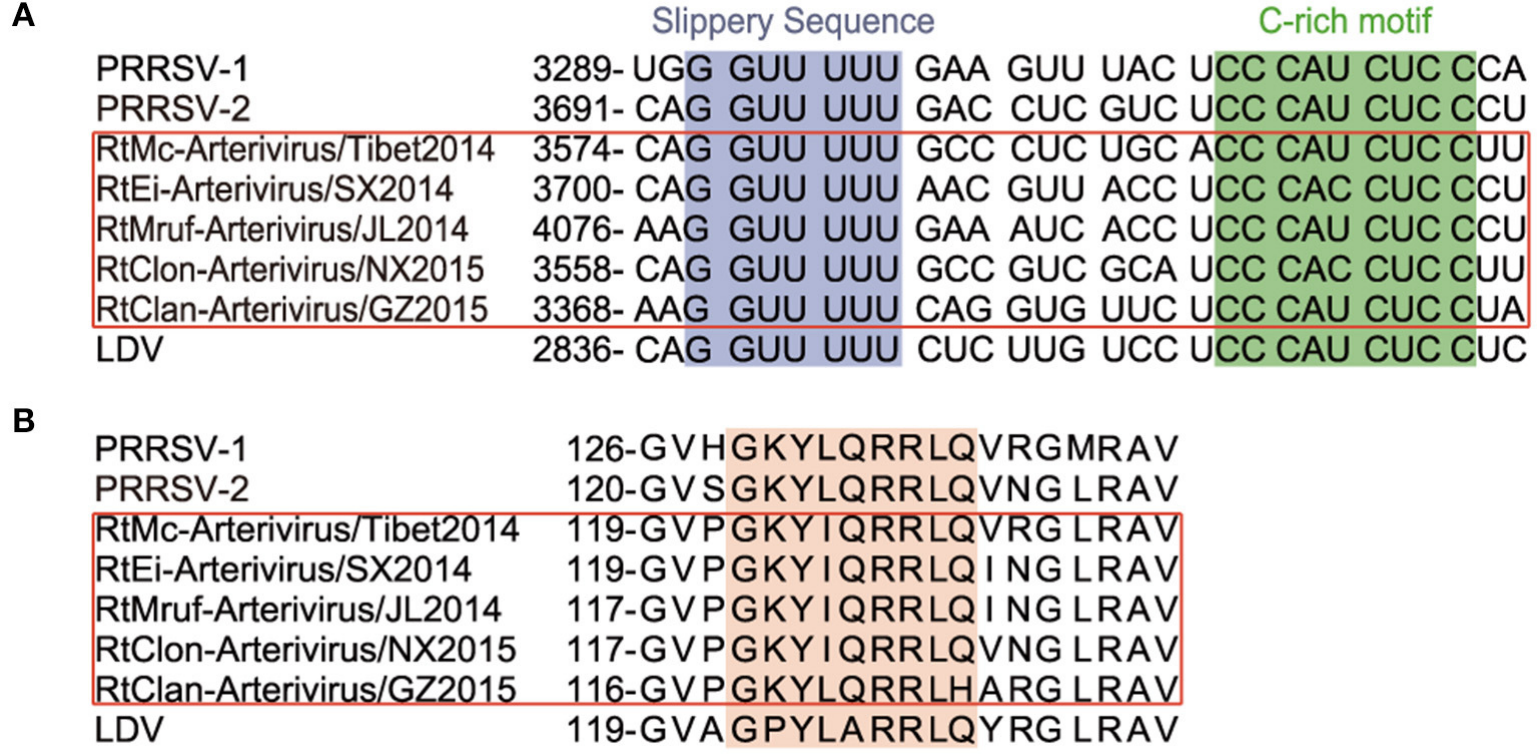
−2 PRF-related elements of arterivirus. The conserved “slippery sequence” and C-rich motif in nsp2 are highlighted in purple and green, respectively **(A)**, and the transactivated protein sequence in nsp1β is highlighted in orange **(B)**.

All members of arterivirus except EAV use a −2 programmed ribosomal frameshifting (−2PRF) mechanism and produce a transframe (TF) protein (nsp2TF) at an efficiency of about 20% of nsp2. This protein, comprising the N-terminal two thirds of nsp2 fused to a 169-aa fragment encoded by the TF ORF, is stimulated by a highly conserved “slippery sequence” (G_GUU_UUU) and a C-rich motif (CCCANCUCC) 11 nt-downstream. Moreover, it was identified that a subunit of viral protein nsp1β (GKYLQRRLQ) is a transactivator of efficient −2 PRF expression (24). We found that the nsp2 of rodent arteriviruses also has the conserved slippery sequence as well as the C-rich motif mentioned above, and also contains the transactivation protein sequence in nsp1β, which is assumed to be able to perform −2PRF for production of nsp2TF (Figure 2).

The TRS (transcription regulatory sequence) is particularly important for viral transcription and replication, especially in the process of discontinuous transcription of subgenomic mRNA. The genomic TRS of rodent arterivirus can be summarized as [U/A]UAACC, which is similar to the UUAACC of PRRSV and UAUAACC of LDV (Supplementary Table S2). Although the base composition of the first four positions in the TRS varies to some extent, the highly conserved CC at the last two positions is critical for complementary base pairing during discontinuous transcription. We also found that although these TRS sequences differ between the ORFs, they all have a certain degree of conservation in the same ORF, except for RtClan-Arterivirus/GZ2015 in ORF5 and RtMc-Arterivirus/Tibet2014 in ORF6.

### Codon usage bias

Analysis of codon usage of the rodent arterivirus genomes revealed a slightly lower proportion of A (21.62%) compared with U (26.09%), G (25.69%), and C (26.60%) (Supplementary Table S3). The ENC values of all 43 arteriviruses were >45, which implies that their codon usage bias is weak (Supplementary Table S3). CAI can be used to assess the expression level of genes, generally between 0 and 1, with larger values indicating a stronger preference for codon use, and the CAI of these 43 arteriviruses were all below 0.23, again indicating that their codon usage bias is weak (Supplementary Table S3).

**Figure 3:**
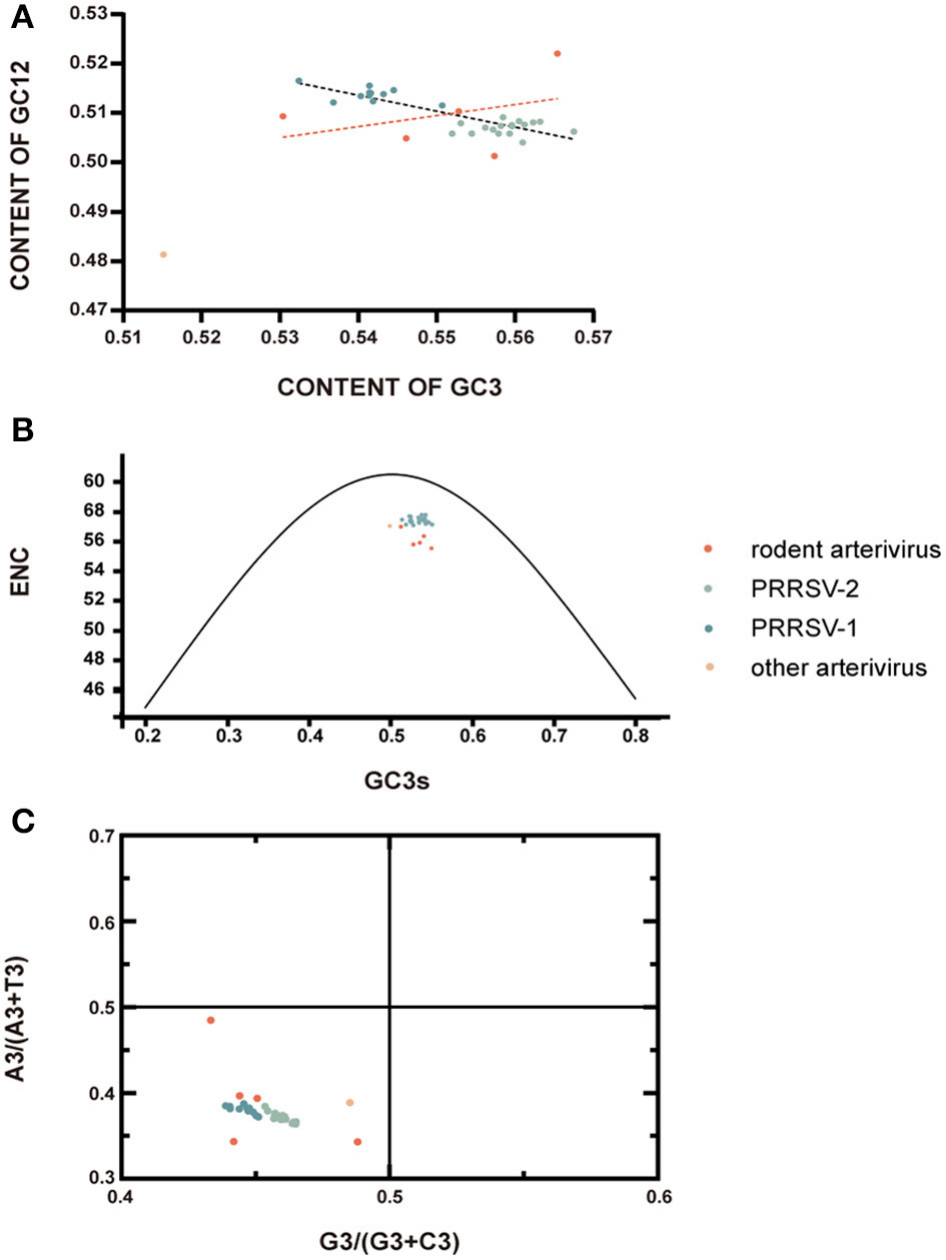
Selection pressure analysis. **(A)** Neutrality plot analysis. GC12 plotted against GC3. The dashed black line is fitted by the scatter points of GC12 and GC3 of PRRSV, and the black formula next to it illustrates the relationships between them; rodent arterivirus is represented by the orange line. **(B)** ENC plotted against GC3s. The black curve represents the theoretical ENC values when the codon usage was only determined by the GC3s composition. **(C)** PR2-plot analysis. A3/(A3 + U3) is plotted against G3/(G3 + C3); the midpoint represents A = U and G = C.

Neutrality plot analysis showed that the regression coefficient between GC3 and GC12 of rodent arterivirus is 0.2217, with a correlation coefficient of 0.3740, meaning the correlation was not significant (Figure 3A). Their coefficient of determination was 0.1399; i.e. only 13.99% change of GC3 resulted in a change of GC2 (Figure 3A). All in all, these data show that the base composition of the codon at the first and second position is quite different from that of the third position. Moreover, the codon usage bias of the rodent arteriviruses is more affected by the pressure of directional mutations of external natural selection than the pressure of their own non-directional mutations. The regression coefficient and the correlation coefficient between GC3 and GC12 of PRRSV were −0.3235 and −0.8703, respectively, a significant inverse correlation that indicates the base composition of the codon at the first and second position is also quite different from the third. However, the overall GC content was relatively stable, and the own non-directional mutation pressure has a great impact on codon usage bias (Figure 3A).

The mean ENC values for rodent arterivirus and PRRSV were 56.14 and 57.42, respectively, which indicates the codon usage bias in these viruses is a little low. All of the rodent arteriviruses and PRRSV fell far below the standard curve in the ENC-GC3s plots, demonstrating that the codon usage patterns of the two viruses are also affected by external natural selection pressure and other factors (Figure 3B).

PRRSV and the rodent arteriviruses were all distributed in the lower left area of the PR2-plot analysis, indicating that the frequency of nucleotide U and C at the third positions is greater than A and G, evidence of a clear preference (Figure 3C). Therefore, it can be inferred that the usage patterns of these codons are not only affected by their self-mutations, but also by factors such as natural selection.

**Figure 4:**
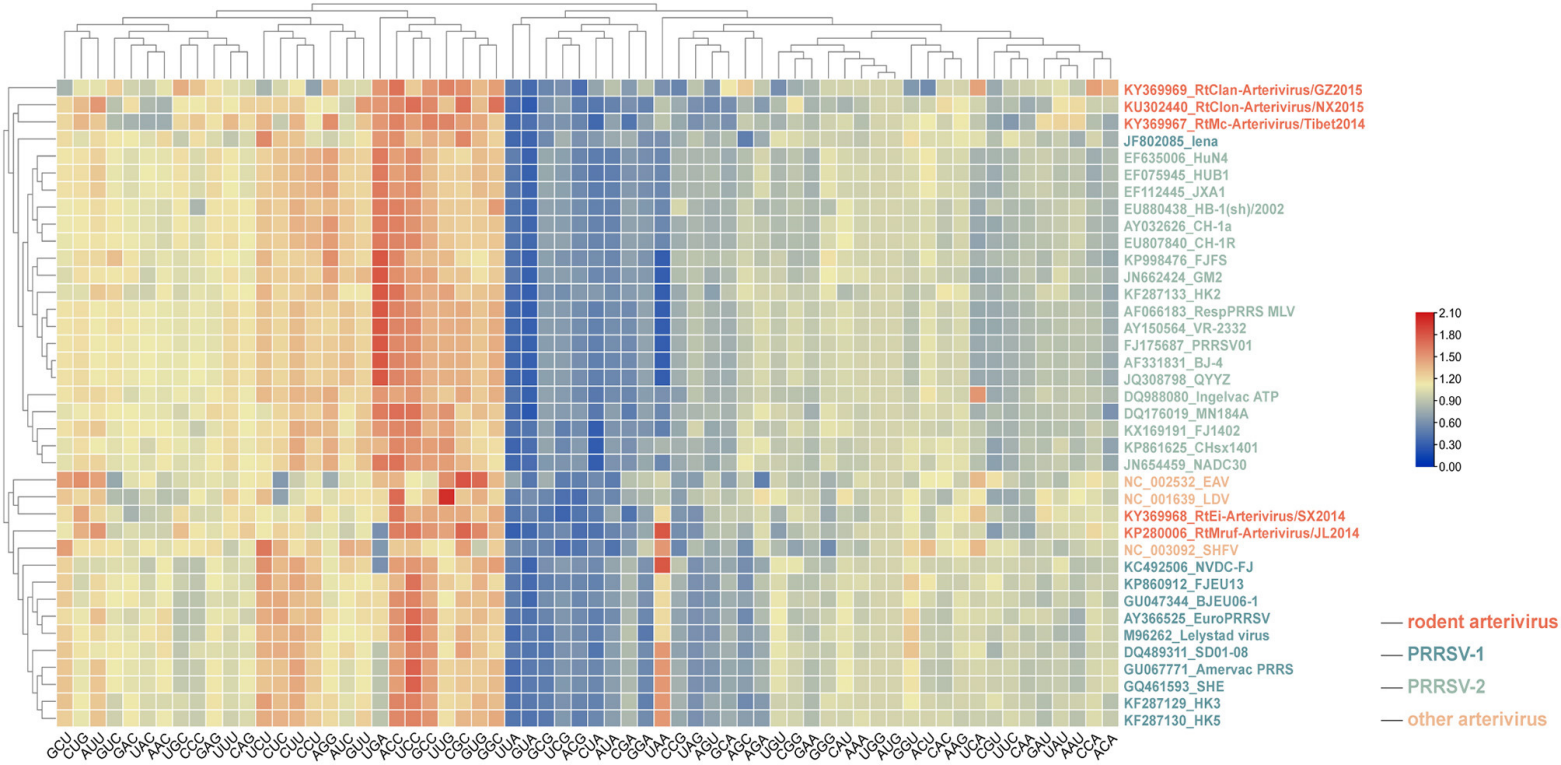
Relative synonymous codon usage (RSCU) comparisons between different arteriviruses. Frequently used codons with higher RSCU values are in red, moderately used codons are in yellow, while the less frequently used codons are represented in blue.

As shown in Figure 4, the codon usage pattern of rodent arterivirus is very similar to that of other arteriviruses. RtClan-Arterivirus/GZ2015, Arterivirus/NX2015 and RtMc-Arterivirus/Tibet2014 clustered with PRRSV-2, RtMruf-Arterivirus/JL2014 clustered with PRRSV-1, and RtEi-Arterivirus/SX2014 was similar to LDV (Figure 4).

### Phylogenetic analysis and evolutionary analysis

**Figure 5:**
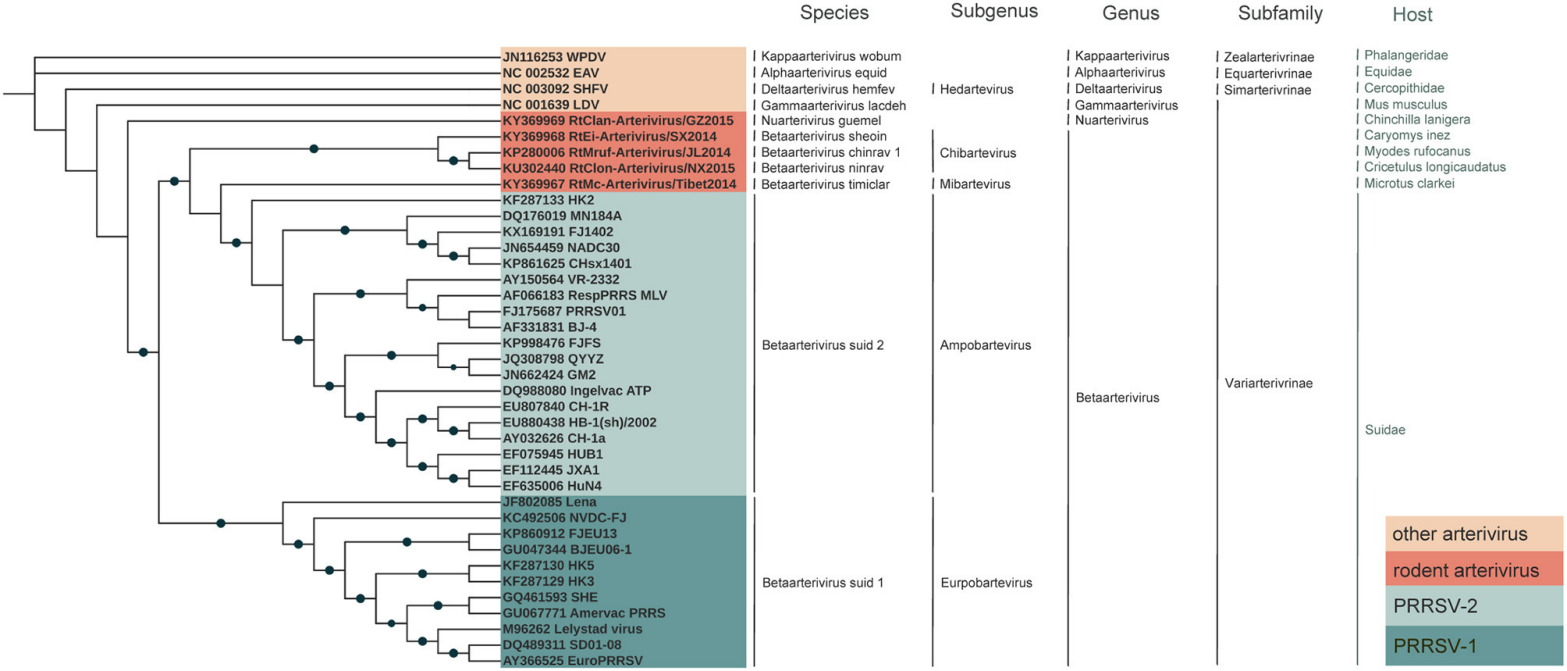
Phylogenetic analyses of arterivirus. Whole genome trees were constructed by using the maximum-likelihood (ML) method and bootstrap values calculated from 1,000 trees.

Phylogenetic trees of the rodent arteriviruses, PRRSV and other arteriviruses were constructed based on nucleotide sequences of the full genome or RdRp, Hel, 3CLpro, and N genes. In the full genome-based phylogenetic tree, RtMc-Arterivirus/Tibet2014, RtEi-Arterivirus/SX2014, RtMruf-Arterivirus/JL2014, and RtClon-Arterivirus/NX2015 clustered together with PRRSV-2 and were closer than PRRSV-1, while RtClan-Arterivirus/GZ2015 was outside these strains (Figure 5).

RtMruf-Arterivirus/JL2014, RtEi-Arterivirus/SX2014, RtClon-Arterivirus/NX2015, RtMc-Arterivirus/Tibet2014 and PRRSV all belong to *Betaarterivirus*, while RtClan-Arterivirus/GZ2015 belongs to another genus, *Nuarterivirus* (Figure 5). In trees based on the three highly conserved RdRp, Hel and 3CLpro genes, it can be seen that RtMc-Arterivirus/Tibet2014, RtEi-Arterivirus/SX2014, RtMruf-Arterivirus/JL2014, Arterivirus/NX2015 were closer to PRRSV-2 than PRRSV-1, but RtClan-Arterivirus/GZ2015 and RtDs-Arterivirus-4/IM2014 were always on the periphery (Supplementary Figures S1A–C). However, these strains were relatively close to PRRSV-1 in the N-based tree (Supplementary Figure S1D).

**Figure 6:**
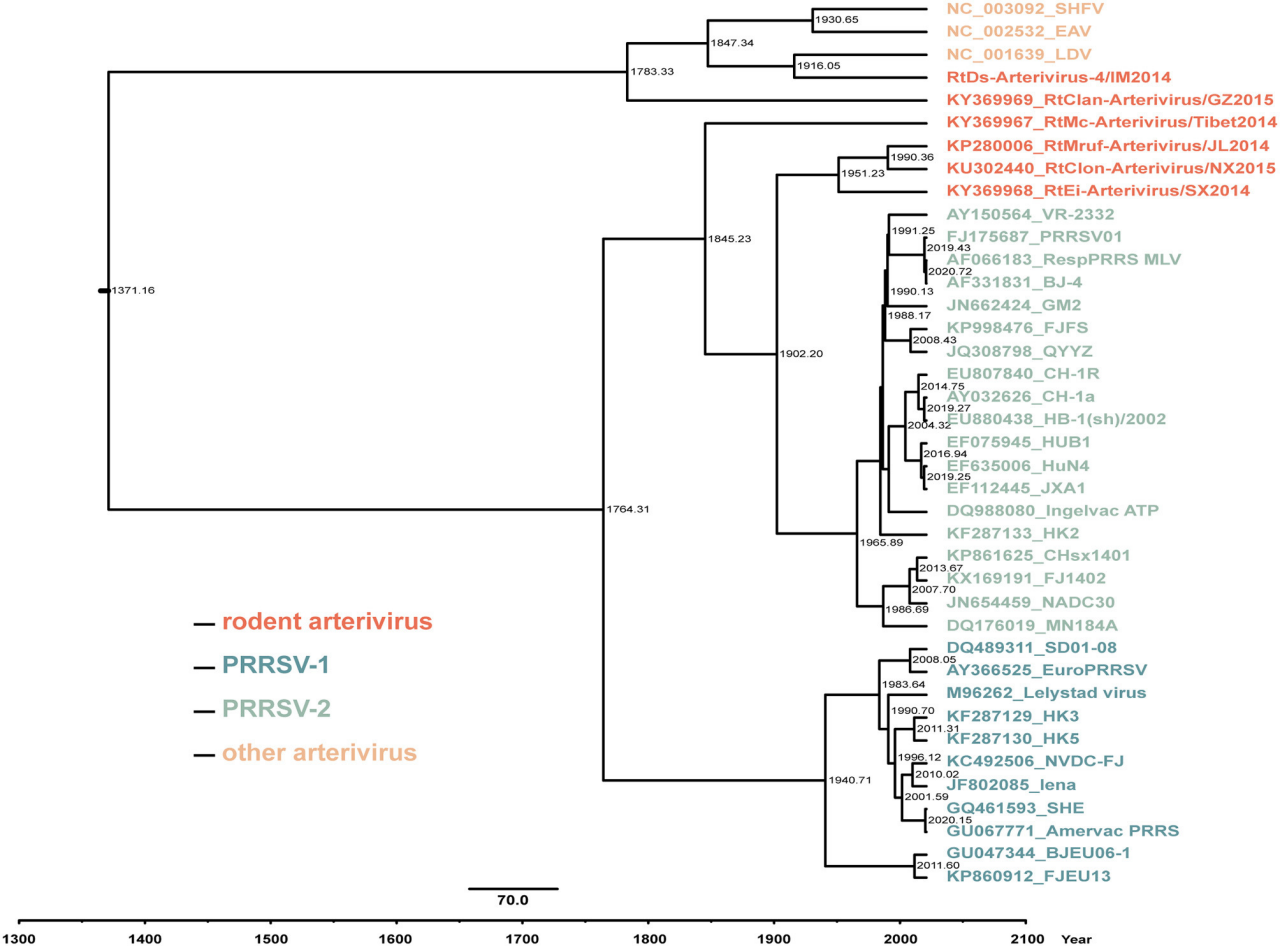
Estimation of the mean time to the most recent common ancestor (tMRCA) for arterivirus based on the helicase (Hel) gene. The time-scaled phylogeny was summarized from all Markov chain Monte Carlo (MCMC) phylogenies of the Hel gene data set analyzed under the GTR + F + G4 nucleotide substitution model and the uncorrelated lognormal relaxed constant clock model in BEAST v1.10.4. The numbers near the internal node indicate the estimated time of divergence.

The results of molecular clock analysis using the Hel gene were consistent with the phylogenetic tree, with RtEi-Arterivirus/SX2014, RtMruf-Arterivirus/JL2014, and RtClon-Arterivirus/NX2015 closest to PRRSV-2, followed by RtMc-Arterivirus/Tibet2014, RtClan-Arterivirus/GZ2015 and RtDs-Arterivirus-4/IM2014 at the periphery of PRRSV (Figure 6). In addition, we found that the mean time to the most recent common ancestor (tMRCA) of RtEi-Arterivirus/SX2014 was March 1951 [95% highest posterior density (HPD), April 1853 to July 2006], that of RtMruf-Arterivirus/JL2014 and RtClon-Arterivirus/NX2015 was May 1990 (95% HPD, October 1930 to February 2018), that of RtMc-Arterivirus/Tibet2014 was March 1845 (95% HPD, April 1614 to October 1957), that of LDV and RtDs-Arterivirus-4/IM2014 was January 1916 (95% HPD, April 1643 to February 2014), and that of RtClan-Arterivirus/GZ2015 was April 1783 (95% HPD, December 1406 to November 1982) (Figure 6). It can be seen that RtEi-Arterivirus/SX2014, RtMruf-Arterivirus/JL2014, and RtClon-Arterivirus/NX2015 appeared earlier than PRRSV and later than LDV, and not only was RtClan-Arterivirus/GZ2015 in the periphery of PRRSV, but it also appeared much earlier than LDV.

### Nucleotide, amino acid identity and codon usage analysis

**Figure 7:**
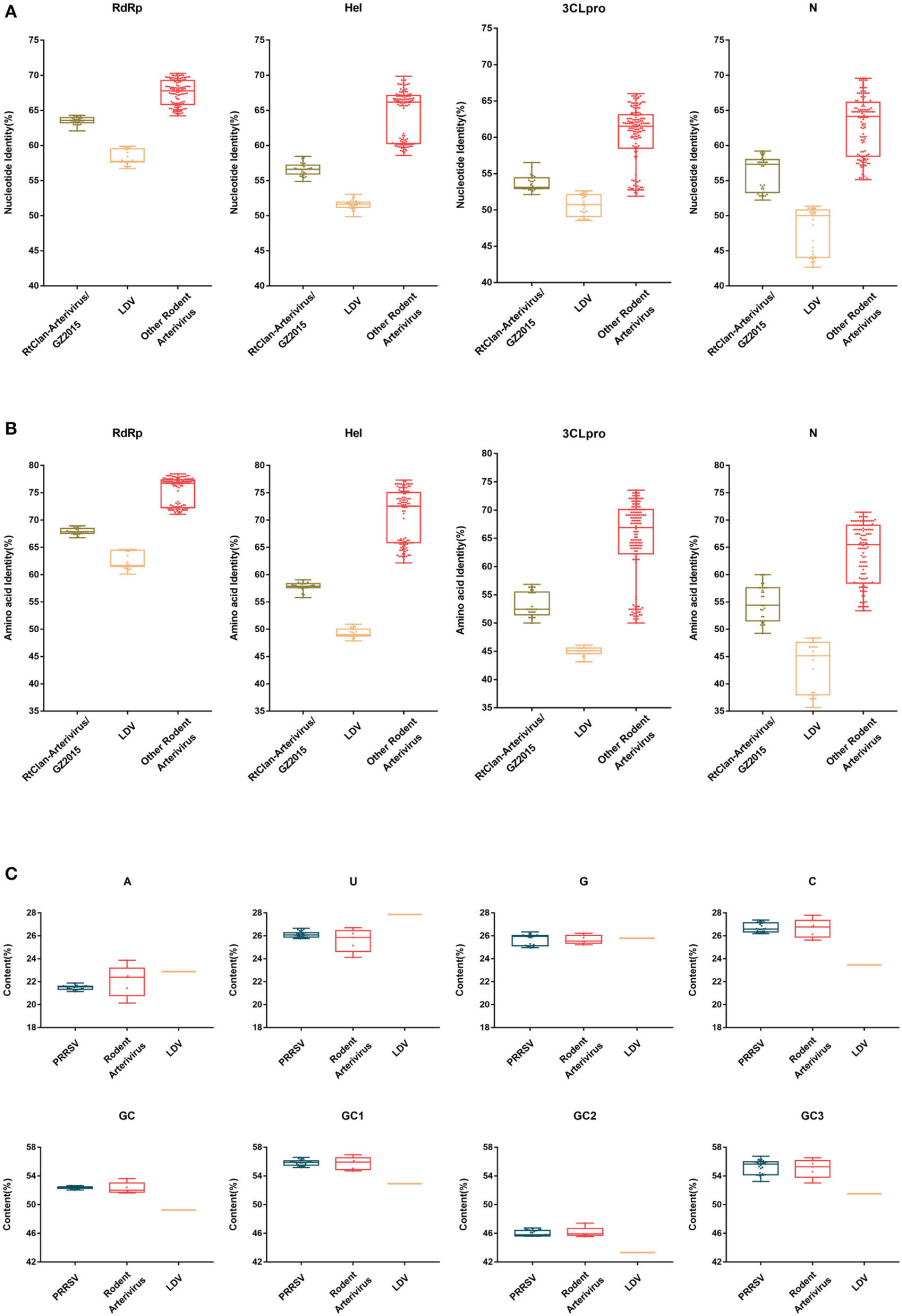
Nucleotide, amino acid identity and codon usage bias of PRRSV and rodent arterivirus. The nucleotide identity **(A)** and putative amino acid identity **(B)** compared to PRRSV of RNA-dependent RNA polymerase (RdRp), helicase (Hel), 3C-like protease (3CLpro) and nucleocapsid (N) genes were analyzed by BioAider v 1.314. The A%, U%, G%, C%, GC1, GC2, GC3, and GC of arterivirus **(C)** were calculated using the CAI calculator website (http://genomes.urv.es/CAIcal/). Each dot represents the corresponding value of each strain. The box size represents the interquartile range (IQR), which is equal to the difference between the upper quartile and the lower quartile. The middle horizontal line of the box represents the median of the samples.

The nucleotide and amino acid identity of PRRSV relative to RtClan-Arterivirus/GZ2015, LDV and other rodent arteriviruses in RdRp, Hel, 3CLpro and N is shown in Figure 7. The identity of PRRSV was clearly more similar to the rodent arteriviruses than to LDV, and the average difference in identity values was about 10–21% (Figures 7A, B), indicating that rodent arteriviruses have a closer evolutionary relationship with PRRSV than LDV. However, RtClan-Arterivirus/GZ2015, which has a more distant evolutionary relationship, is more similar than LDV to PRRSV, inconsistent with the evolutionary tree and molecular clock analysis.

Since RtClan-Arterivirus/GZ2015 was closer to rodent arterivirus at the nucleotide level, we analyzed codon usage of all the rodent arteriviruses. Based on the analysis, the GC content of codons in three positions is different, and the average GC content at each position (high to low) was GC1, GC3, GC2.We can also see that PRRSV and rodent arteriviruses are very similar, but there is a gap with LDV, indicating that the codon usage bias of PRRSV and rodent arterivirus is more similar than LDV (Figure 7C).

## Discussion

In this article, we collected sequences from 7 rodent arteriviruses, 33 PRRSV isolates, and three other representative arteriviruses (LDV, SHFV and EAV) and conducted in-depth analysis of their genomes, codons, and phylogenies. The newly discovered rodent arteriviruses have a genome size and organization similar to other arteriviruses, and share other conserved features such as slippery sequences and C-rich motifs in the nsp2, and the transactivated protein sequence in nsp1β. In addition, the TRS of these viruses is very similar to that of PRRSV and LDV, indicating a common transcription regulatory strategy and supporting their close evolutionary relationship.

We found all 43 arteriviruses studied to have weak codon usage bias and are under greater pressure from external natural selection than from their own non-directional mutations. Although the primary structure of proteins obtained after translation from synonymous codons is the same, codon usage bias may affect gene expression, as reflected in the effects on mRNA secondary structure and protein abundance (25, 26). Therefore, the similarity of codon usage bias between PRRSV and rodent arteriviruses suggests that there are similar gene expression patterns between them, further evidence of a close evolutionary relationship.

From evolutionary analysis, it can be concluded that PRRSV diverged from other arteriviruses more than 100 years ago, and PRRSV likely existed in swine prior to its discovery in the late 1980s (6). Previous studies speculated that because LDV is older and closest to PRRSV in the *Arteriviridae*, some LDV-like rodent virus might spread to wild boars in Asia and Europe through wounds and other routes, making them intermediate hosts (27, 28). According to this theory, PRRSV-1 was transmitted from European wild boars to European domestic swine, and PRRSV-2 would have been transmitted to American domestic pigs through wild boars imported into America (27). However, no strong evidence has been found for the presence of this type of LDV-like rodent virus in wild boars until now, making the origin of PRRSV untraceable (29) until now.

Through phylogenetic and origin analysis combined with the tMRCA of these arteriviruses, we speculate that an early RtClan-Arterivirus/GZ2015 was likely transmitted from *Chinchilla lanigera* to *Microtus clarkei* in Tibet to form RtMc-Arterivirus/Tibet2014, then might spread to the *Circetidae* in Shanxi, Jilin and Ningxia to form several similar strains. Finally, these *Circetidae* were possibly transported to Europe and the Americas to infect domestic pigs, forming the two types of PRRSV. It is worth noting that in the 20th century, many countries worldwide had not form a large-scale pig industry, pigs were usually kept free-range; therefore, rats or other rodent species could circulate freely in the pigsty. Pig feed might be contaminated with rodent feces containing certain rodent arteriviruses, which was then eaten by pigs, thus leading to arterivirus cross-species transmission from rodents to pigs. This explanation of PRRSV origin is distinct from other theories (that LDV-infected European wild boars transmitted virus to domestic pigs in Europe and the Americas).

The similarity between PRRSV and rodent arteriviruses is not very high, so there must have been other viruses involved in the evolution of PRRSV, but sequences are currently unavailable for any such intermediate ancestor.

In summary, based on an in-depth evolutionary analysis of the genomes and codon usage bias of the newly discovered rodent arteriviruses, we propose them as novel ancestors of PRRSV. The results fill the gaps in knowledge about the origin and evolution of PRRSV and rodent arteriviruses, and elucidate important insights for monitoring potential interspecies transmission between wild rodents and pigs in the future.

## Data availability statement

The original contributions presented in the study are included in the article/Supplementary material, further inquiries can be directed to the corresponding authors.

## Author contributions

Y-WH, BW, and Z-YZ conceived and designed the study and wrote the paper. Z-YZ, DY, C-MJ, and QZ performed the experiments and analyzed the data. All authors contributed to the article and approved the submitted version.
