# Supplementary material for: Comparative analysis of newly identified rodent arteriviruses and porcine reproductive and respiratory syndrome virus to characterize their evolutionary relationships
Source: Front Vet Sci. 2023 Apr 3;10:1174031. doi: 10.3389/fvets.2023.1174031 (PMC10106604; doi:10.3389/fvets.2023.1174031)
Supplement: Supplementary file 1 [file Data_Sheet_1.DOCX]

Supplementary Material

Comparative analysis of newly identified rodent arteriviruses and porcine reproductive and respiratory syndrome virus to characterize their evolutionary relationships

**Zhuangyan Zhao^1^, De Yu^1^, Chun-Miao Ji^2^, Qiankun Zheng^3^, Yao-Wei Huang^1,2*^, Bin Wang^2*^* Correspondence:**

email@uni.edu*Yao-Wei Huang, Zhejiang University, Hangzhou, 310058, Zhejiang.

Email: [yhuang@zju.edu.cn](mailto:yhuang@zju.edu.cn)

*Bin Wang, Guangdong Laboratory for Lingnan Modern Agriculture, College of Veterinary Medicine, South China Agricultural University, Guangzhou, 510642, China

E-mail: [hlab-wangbin@zju.edu.cn](mailto:hlab-wangbin@zju.edu.cn)

**Supplementary Figure 1.** Gene-based phylogenetic analyses of arterivirus. Trees based on RNA-dependent RNA Pol (RdRp) (**A**), helicase (Hel) (**B**), 3CLpro (**C**), and nucleocapsid (N) (**D**) were constructed by using the maximum-likelihood (ML) method and bootstrap values calculated from 1,000 trees.

**Supplementary Table 1.** Comparison of nucleotide and amino acid identities and other genomic features of rodent arterivirus, PRRSV-1, PRRSV-2 and other representative arteriviruses.

**Supplementary Table 2.** Putative transcription regulatory sequences of rodent arterivirus genomes.

**Supplementary Table 3.** Basic arterivirus codon information.
