# Supplementary figures and images for: Comparative analysis of newly identified rodent arteriviruses and porcine reproductive and respiratory syndrome virus to characterize their evolutionary relationships
Source: Front Vet Sci. 2023 Apr 3;10:1174031. doi: 10.3389/fvets.2023.1174031 (PMC10106604; doi:10.3389/fvets.2023.1174031)

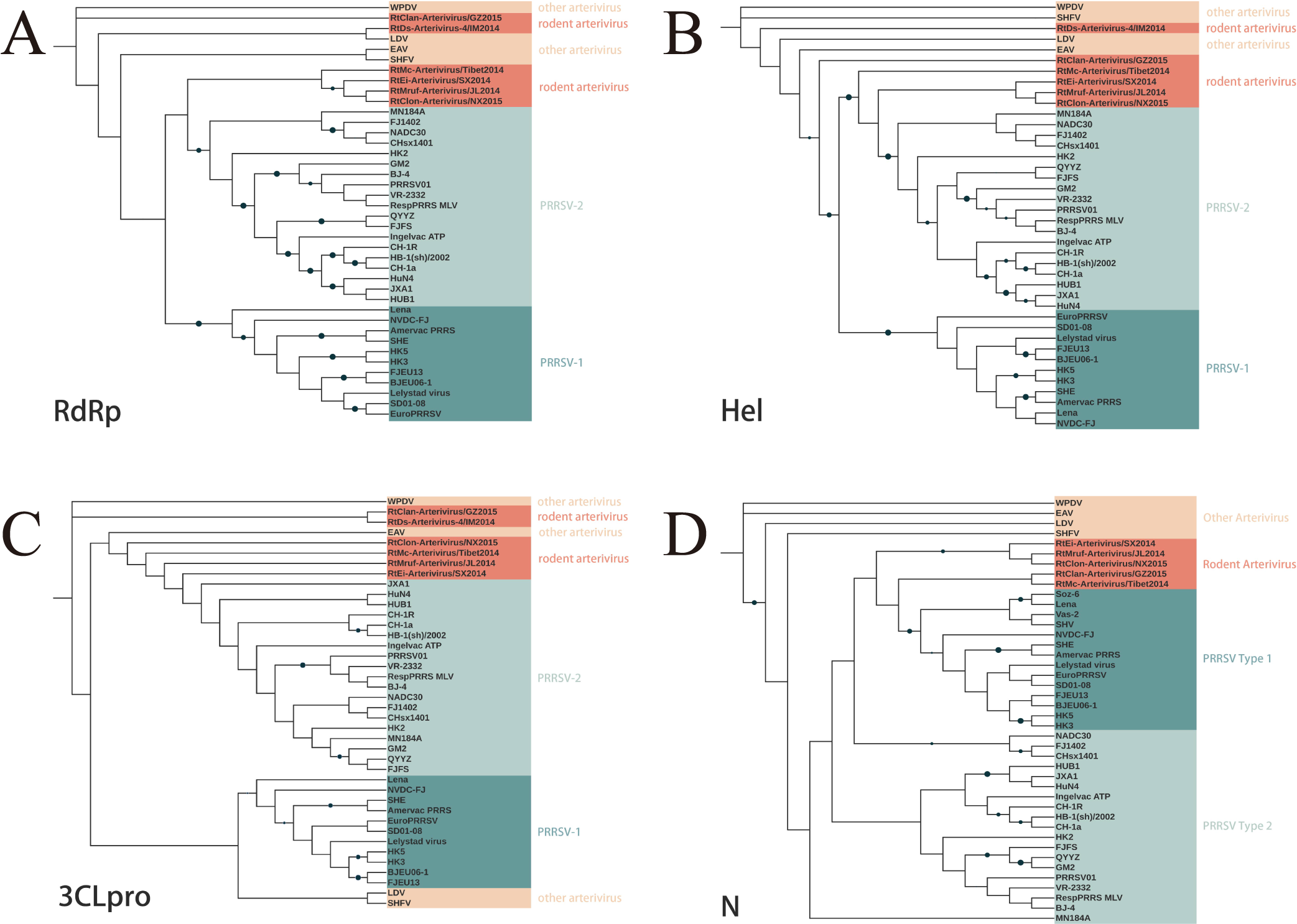

Supplement: Supplementary file 5 [file Image_1.TIF]
